# Non-canonical non-genomic morphogen signaling in anucleate platelets: a critical determinant of prothrombotic function in circulation

**DOI:** 10.1186/s12964-023-01448-y

**Published:** 2024-01-03

**Authors:** Paresh P. Kulkarni, Mohammad Ekhlak, Debabrata Dash

**Affiliations:** Center for Advanced Research on Platelet Signaling and Thrombosis Biology, Department of Biochemistry, Institute of Medical Sciences, Banaras Hindu University, Varanasi, 221005 Uttar Pradesh India

**Keywords:** Platelet activation, Notch, Sonic Hedgehog (Shh), Wnt, Non-canonical signaling, Vismodegib

## Abstract

Circulating platelets derived from bone marrow megakaryocytes play a central role in thrombosis and hemostasis. Despite being anucleate, platelets express several proteins known to have nuclear niche. These include transcription factors and steroid receptors whose non-genomic functions are being elucidated in platelets. Quite remarkably, components of some of the best-studied morphogen pathways, namely Notch, Sonic Hedgehog (Shh), and Wnt have also been described in recent years in platelets, which regulate platelet function in the context of thrombosis as well as influence their survival. Shh and Notch pathways in stimulated platelets establish feed-forward loops of autocrine/juxtacrine/paracrine non-canonical signaling that helps perpetuate thrombosis. On the other hand, non-canonical Wnt signaling is part of a negative feedback loop for restricting platelet activation and possibly limiting thrombus growth. The present review will provide an overview of these signaling pathways in general. We will then briefly discuss the non-genomic roles of transcription factors and steroid receptors in platelet activation. This will be followed by an elaborate description of morphogen signaling in platelets with a focus on their bearing on platelet activation leading to hemostasis and thrombosis as well as their potential for therapeutic targeting in thrombotic disorders.

## Introduction

Development of multicellular tissues and organs with unique morphology from single-celled zygote has long fascinated scientists. Each cell in a fully developed organism has the same set of genes as the zygote has, yet the organism is composed of a multitude of cell types that differ in their forms, functions, and locations within the body. This would require temporally and spatially orchestrated expression of specific set of genes during development. Alan Turing, a polymath more renowned for his work in computer science, first theorized in 1952 a chemical basis for this morphogenesis [1]. He coined the term “morphogen” for the proposed chemical entities that are responsible for emergence of shapes and patterns from cells to form tissues and organs. The concept was way ahead of its time, and it took more than three decades for the first morphogen Bicoid to be discovered by Christiane Nusslein-Volhard in 1988 [2].

**Fig. 1:**
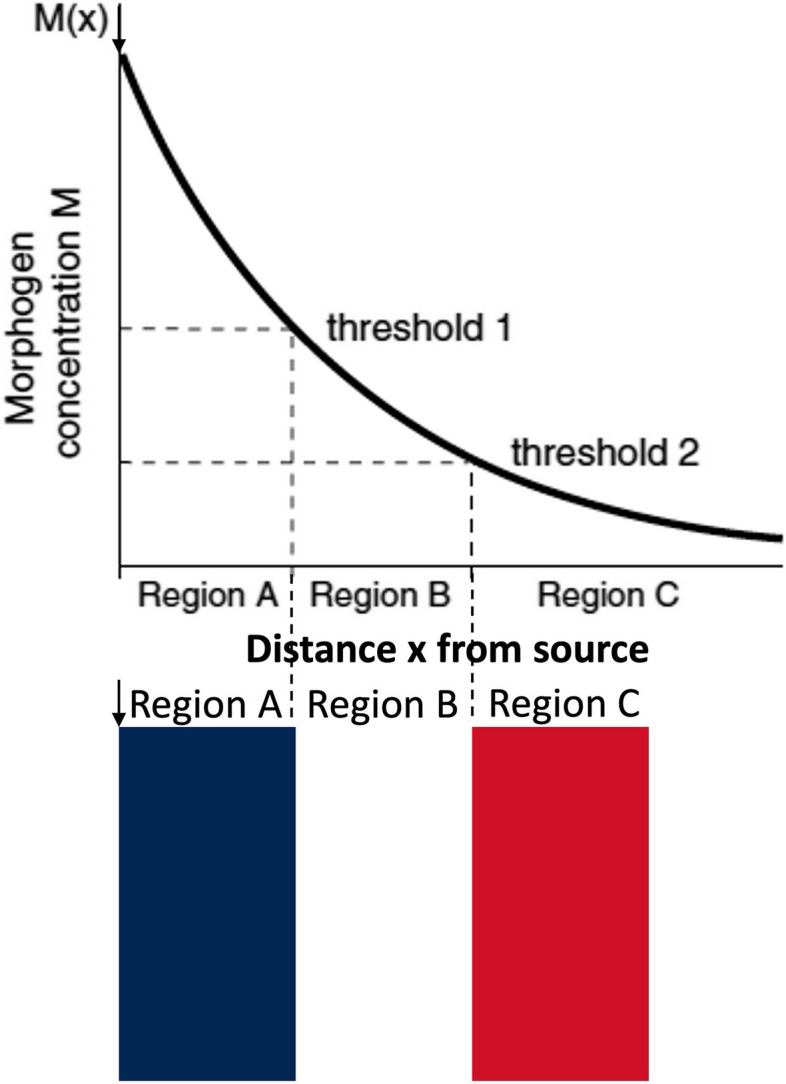
The French-Flag model demonstrating the mechanism of action of morphogens. Upper panel, graph showing exponential decline in morphogen concentration M (Y-axis) with distance x from the source (X-axis). Lower panel, the French flag illustration demonstrating differential responses of cells along the gradient of morphogen originating from the source cell (indicated by arrow) and diffusing towards target cells located in three different regions located at varying distances from the source

Morphogen is generally defined as a molecule released from a source cell that wields specific biological effects on a remotely positioned target cell, such that the quantum and nature of its effects are governed by morphogen concentration prevalent at the location of target cell [3]. The most simple “*French Flag*” model for mechanism of action of morphogens was proposed by Lewis Wolpert in 1969 [4] and championed by the *Drosophila* biologist Peter Lawrence in his book “*The Making of a Fly*” in 1992 [5]. The model predicts emergence of varying patterns / regions of cells driven by exponentially decreasing gradient of morphogen owing to diffusion (Fig. 1).

Cells in Region A (corresponding to blue sector in the French flag) that are closer to the source of morphogen (indicated by arrow) express a certain set of genes with either a high (threshold 1) or low threshold (threshold 2) for morphogen concentration to elicit a response. Cells at an intermediate distance from the source (Region B in white) have a different gene expression profile in response to the same morphogen as only the genes with low threshold (threshold 2) for morphogen concentration are expressed. The farthest cells (Region C depicted in red) exposed to morphogen concentration lower than the threshold 2 do not respond to the morphogen. The model can be extended to either 2- or 3-dimensional gradients as well as to more than 3 regions. However, this simple model has several caveats, the most important being the vulnerability to even small fluctuations in production rates of morphogen and, secondly, the model fails to account for mechanisms to remove morphogen towards the end of morphogen range of action, which is pivotal to maintaining the gradient. A model that addresses these limitations has been proposed where a component of self-enhanced degradation/removal of morphogen that is proportional to the power of morphogen concentration is included to yield a power-law gradient [3]. Thus, changes in morphogen concentrations at source result in proportionate changes in the boundaries of regions without affecting the relative sizes of the regions. Such a model is more robust to changes in morphogen generation rates from source.

Several of the morphogens were first recognized through *Drosophila* mutants with characteristic developmental defects, which has inspired the nomenclature of morphogen signaling pathways. For example, Notch derives its name from the notched wings of flies with mutations in the gene for Notch receptor [6], while the name Hedgehog was inspired from a patch of disorganized hair-like bristles on mutants that resemble the hedgehog spines [7]. Along the same lines the gene encoding Wnt proteins is named Wingless [8]. Morphogens evolved very early during evolution of multi-cellular organisms and most components of morphogen signaling pathway are evolutionarily conserved to even humans, possibly attributable to their stellar fundamental roles in morphogenesis. Loss of morphogen function during development can be profoundly detrimental. For example, disruption of Hedgehog signaling can result in holoprosencephaly [9], a developmental disorder with brain and facial abnormalities while defective Notch signaling can lead to severe vertebral column anomalies such as spondylocostal dysostosis [10]. There has been an explosion of information on signaling mechanisms adopted by individual morphogens and their cellular outputs. Although the role of morphogens during development has been well-characterized, their expression as well as function during various physiological and disease states in adult organisms is only being recently unraveled.

Platelets are circulating blood cells derived from bone marrow megakaryocytes that serve a critical purpose of forming clots to prevent blood loss following vascular injury. A similar but more exaggerated response from platelets underlies arterial thrombosis and its potentially fatal consequences such as acute myocardial infarction (AMI) or ischemic stroke [11]. Despite being enucleate, platelets synthesize proteins from mRNA transcripts inherited from megakaryocytes [12], carry machinery for processing of mRNA [13, 14] and can even generate progeny [15]. Intriguingly, platelets express several molecules known to have overarching genomic niche that include transcription factors and steroid receptors whose functions are not clearly delineated [16, 17]. Quite remarkably, components of some of the best-studied morphogen pathways, namely Sonic Hedgehog (Shh) [18], Wnt [19] and Notch [20], have also been described in recent years in platelets, which pursue non-canonical, non-genomic signaling routes integral to platelet physiology and thrombogenicity, and can be promising therapeutic targets. The present review aims to provide an overview of these signaling pathways in general, with focus on their bearing on platelet activation leading to hemostasis and pathological thrombosis.

## Notch signaling

**Fig. 2:**
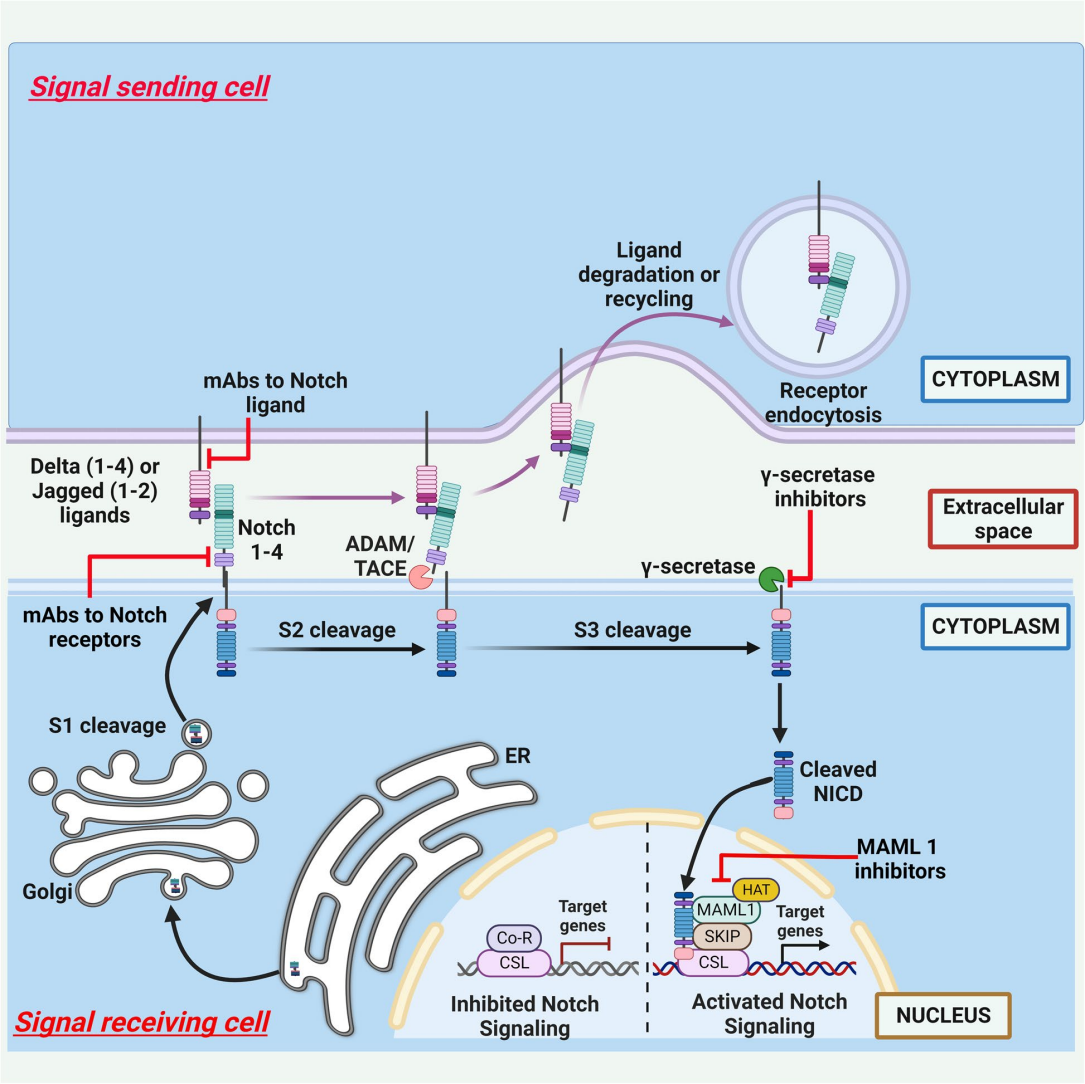
Canonical Notch signaling. Notch receptor is synthesized in the endoplasmic reticulum and gets modified by proteolytic cleavage (S1 cleavage). Notch expressed on the plasma membrane of various cell types interacts with transmembrane ligands such as Delta on the surface of an adjacent cell that leads to a series of proteolytic cleavages of Notch by a disintegrin and metalloproteinase (ADAM) (S2 cleavage), followed by γ-secretase complex (S3 cleavage). The intramembrane nicking of Notch receptor by the latter enzyme liberates a C-terminal fragment of the receptor, namely the Notch intracellular domain (NICD). NICD in turn translocates into the nucleus, where it binds to CSL or RBPJ to regulate expression of various downstream genes by recruiting co-activators such as Mastermind-like (MAML). Notch ligands undergo endocytosis and degradation or recycling back to plasma membrane. Potential therapeutic inhibitors of targets involved in the Notch signaling include monoclonal antibodies (mAbs) targeting the Notch ligands or receptors and small-molecules or mAb inhibitors of γ -secretase

Our understanding of Notch signaling originate from genetic studies in *Drosophila melanogaster* when mutant flies with notched wings were identified by John Dexter and Thomas Hunt Morgan [21]. Additional mutants were identified with different wing phenotypes such as Delta, Serrate and Fringe [22–24]. With the beginning of 1980s using a combination of fly genetics, molecular biology and biochemistry Notch receptor and other components of Notch signaling were gradually discovered revealing a relatively simple architecture. Notch receptor expressed on the plasma membrane of various cell types interacts with transmembrane ligands such as Delta on the surface of an adjacent cell initiating a juxtacrine signaling (Fig. 2) [25]. The receptor-ligand binding leads to a series of proteolytic cleavages of Notch receptor by a disintegrin and metalloproteinase (ADAM), followed by γ-secretase complex. The intramembrane nicking of Notch receptor by the latter enzyme liberates a C-terminal fragment of the receptor, namely the Notch intracellular domain (NICD). NICD in turn translocates into the nucleus, where it binds to CSL (Suppressor of Hairless, Lag-2) or RBPJ (Recombining binding protein suppressor of hairless) to regulate expression of various downstream genes. The resulting output response of the cell is highly varied and depends on the cell type and its environment. Although Notch signaling is juxtacrine in nature some instances of long distance signaling has also been reported through dynamic filopodia [26] or using exosomes [27].

### Notch receptor and ligands

Notch receptor is synthesized in the endoplasmic reticulum and gets modified by proteolytic cleavage [28] as well as O-glycosylation [29] enroute to plasma membrane. Ligand binding to the post-translationally processed Notch receptor on the plasma membrane exerts a pulling force on the hinged negative regulatory region (NRR) domain to expose the cleavage site for ADAM metalloproteases such as tumour necrosis factor-α convertase (TACE) [30]. Subsequently, a ‘regulated intramembranous processing’ (RIP) by γ-secretase complex liberates the NICD [31]. Incidentally, γ-secretase complex is also involved in processing of CD44, Erb-B2 Receptor Tyrosine Kinase 4 (ErbB4) and amyloid-precursor protein (APP) that releases amyloid-β [32]. NICD can either immediately translocate to the nucleus or undergo a complex routing through endosomal compartment [33]. Like Notch receptor, Notch ligands, too, undergo endocytosis and recycling back to plasma membrane, which is necessary for their maturation into functionally competent ligands [34, 35]. These multiple regulatory steps layered over a basic motif is probably responsible for fine tuning of Notch signaling.

### NICD and CSL

Upon nuclear localization NICD [36] associates with the DNA-binding protein CSL, which has a weak consensus binding sequence of C/tGTGGGAA [37, 38]. CSL-NICD interaction leads to recruitment of Mastermind-like (MAML) to a ternary complex [39]. Formation of the NICD-CSL-MAML complex converts CSL from a repressor to an activator [40, 41]. There is evidence to suggest that a balance between CSL complexed with either co-repressors or co-activators determines the expression of genes downstream of Notch signaling [42]. Several post-translational modifications of NICD are known to occur, which may serve to amplify or dampen the Notch signaling [43–47].

### Signaling output in health and dysregulation in disease

Despite a relatively simple molecular design, Notch signaling leads to expression of diverse arrays of transcriptomes depending on the cell type and context [48]. The possible reasons for this diversity in the signaling output may include the following:

- Access to different sets of DNA response elements due to epigenetic states [49] or to different repertoires of co-activators in different cells and contexts
- Different paralogs of Notch receptor or ligands may have specific functions [50]
- Crosstalk with other signaling pathways such as Wnt/beta-catenin/GSK3beta [46, 51], BMP/TGF-β [52], HIF/FIH [53] and Sonic Hedgehog [54]

Notch signaling plays an important role in development of somite-derived organs [55] as well as organs including vasculature [56], heart [57], and hematopoietic system [58]. Notch also plays a role in adult tissue homeostasis, such as control of tip-stalk balance in the endothelium [56]. Notch signaling is dysregulated in several disorders such as pulmonary arterial hypertension [59]. Mutations or non-mutational dysregulation of Notch signaling components have been reported in cancers affecting head and neck [60], lung [61], breast [62], and hematopoietic system [63] among other organs. Several drugs targeting Notch signaling including γ-secretase inhibitors (such as RO4929097) [64] and anti-DLL-4 antibodies (such as demcizumab [65] and enoticumab [66]) are currently under clinical trials for different cancers as monotherapy or in combination with other treatments.

### Non-canonical signaling

There are three subclasses of non-canonical Notch signaling:

- Notch signaling can be induced by ligands other than DLL/Jagged. These alternative ligands include MAGP1/2, YB1, DNER, MB3, contactin1 and DLK1 [67].
- Notch signaling can have cellular effects independent of nuclear CSL. Examples include control of FOXO1 expression in Treg cells [68], activation of NF-κB and PI3K pathways in cervical cancer [69], evasion of apoptosis and induction of EMT in tumor cells [70, 71], all of which are independent of interaction with nuclear CSL.
- CSL activation and consequent influence on gene expression can occur independent of NICD [72]. Viral co-activators such as EBNA2 and 13SE1A [73, 74], as well as transcriptional regulator Ptf1a [72], which is important for development of acinar cell lineage in pancreas and nervous system, can bind to and activate CSL.

## Hedgehog signaling

**Fig. 3:**
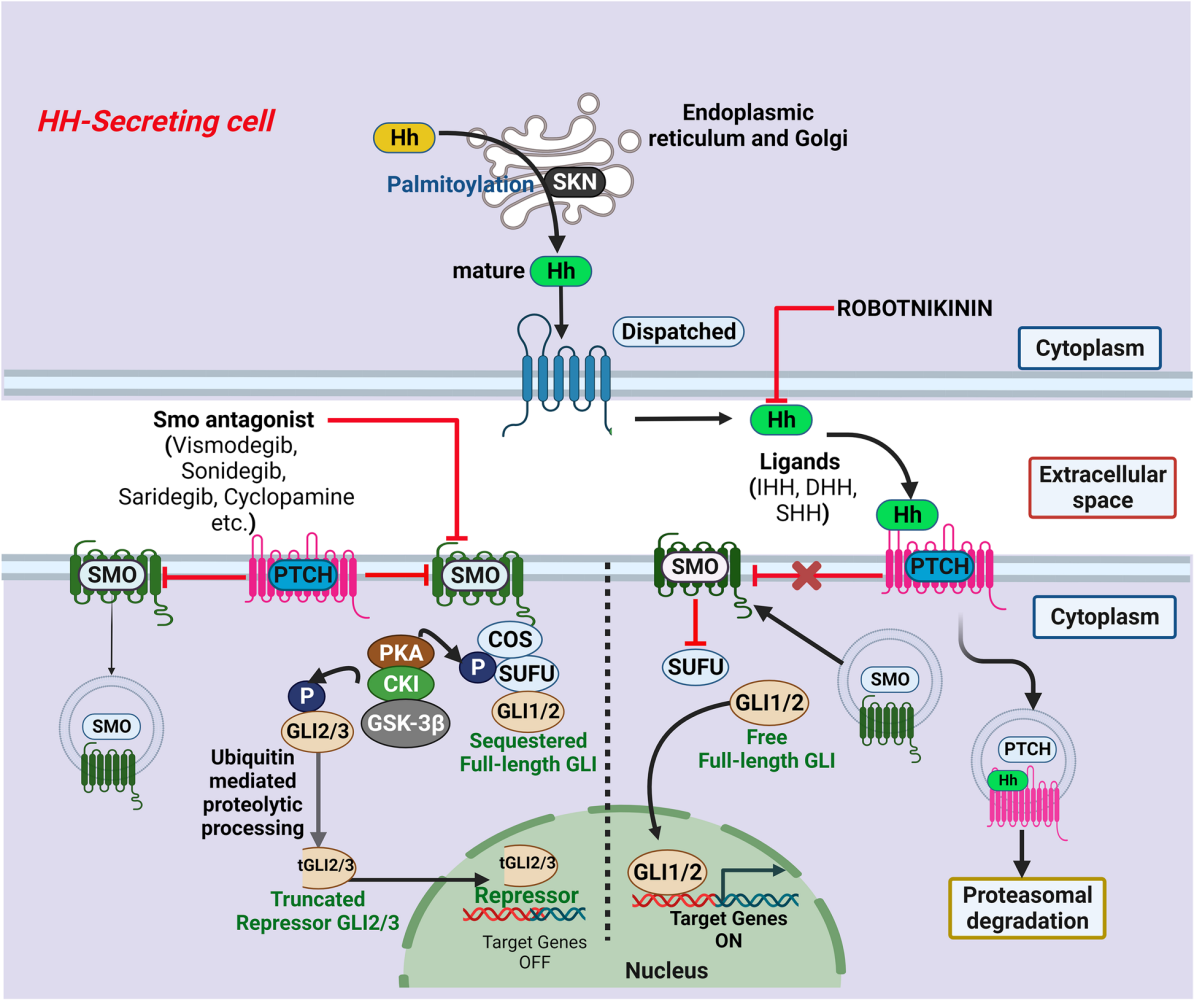
Canonical Hedgehog signaling. The immature full-length HH undergoes autoproteolytic cleavage and is subsequently attached to cholesterol and palmitic acid to form mature HH. The palmitoylation of HH is catalyzed by skinny hedgehog (SKN). Hedgehog (HH) proteins initiate a signaling cascade by binding to Patched (PTCH). In the absence of HH, PTCH binds to and represses Smoothened (Smo), a G-protein coupled receptor (GPCR) by inducing its internalization and degradation and/or cytosolic sequestration of Gli by ‘suppressor of Fu’ (SUFU). HH binding to PTCH leads to its internalization and degradation. This releases Smo from the inhibitory regulation of PTCH. The de-repressed Smo is activated to an open conformation, which signals through G_αi_ to inhibit protein kinase A (PKA), thus preventing post-translational modification and proteolytic processing of Gli family of transcription factors to repressors. Stabilized full-length Gli translocate to the nucleus to upregulate multiple downstream target genes. Potential therapeutic inhibitors of targets involved in the HH signaling include small-molecule antagonists of Smo and HH

Sonic hedgehog (Shh) along with Indian hedgehog (Ihh) and desert hedgehog (Dhh) are the mammalian counterparts of Hedgehog protein [75]. It was first discovered in mutant *Drosophila melanogaster* with characteristic patch of disorganized hair-like bristles that resemble the hedgehog spines [7]. Defect in Shh signaling during development leads to congenital facial and cranial anomalies termed as holoprosencephaly [9]. Shh initiates a signaling cascade [76] by binding to a 12-transmembrane protein cognate receptor Patched (PTCH). In the absence of Shh, PTCH binds to and represses another protein, Smoothened (Smo), a G-protein coupled receptor (GPCR). Shh binding to PTCH leads to internalization and degradation of latter. This releases Smo from the inhibitory regulation of PTCH. The de-repressed Smo is activated to an open conformation, which signals through G_αi_ to activate Gli family of transcription factors. Gli translocates to the nucleus to regulate multiple downstream target genes (Fig. 3) [76].

### Shh

The maturation, secretion and spread of Shh from the source cell is dependent on several post-translational modifications. Upon entering the secretory pathway, the immature full-length Shh undergoes autoproteolytic cleavage by its C-terminal domain to form an N-terminal mature Shh peptide [77]. The Shh peptide thus formed is subsequently attached to cholesterol at its C-terminus [78], as well as linked to palmitic acid through hydroxyl groups in its amino acid side chains [79]. The palmitoylation of Shh is catalyzed by a membrane-bound O-acyl transferase known as ‘skinny hedgehog’ that leads to increased Shh activity [80]. The cholesterol moiety attached to C-terminus sequesters Shh on the plasma membrane restricting its diffusion [81]. Cholesterol-bound Shh oligomerizes [82] at the cell surface, which is essential for its long-range activity. Shh is released from the cell surface by the action of cholesterol-binding proteins such as the transmembrane Dispatched and the soluble SCUBE2 [83], which serve to shield the cholesterol moiety of Shh from the aqueous environment. Secreted Shh then diffuses through tissues as soluble multimers [84], or in association with lipoproteins [85] and extracellular vesicles [86]. In certain instances, Shh associated with cell membrane exerts long-range effects through formation of dynamic filopodial membrane extensions [87].

### PTCH

In the absence of Shh ligand, the PTCH receptor remains bound to Smo that keeps Shh signaling constitutively inhibited [88]. The mechanism of inhibition of Smo by PTCH might involve binding and transport of oxysterols through its sterol-sensing domain (SSD) [89]. Oxysterols are putative Smo ligands [90] that are proposed to induce rapid degradation or intracellular sequestration of Smo [91]. Upon Shh binding PTCH is internalized and degraded. In the absence of PTCH-bound oxysterols, Smo stabilizes and accumulates on the plasma membrane [91]. PTCH binding to Shh is facilitated through interactions between PTCH SSD and Shh-associated cholesterol [92]. In addition, evidence suggests that Shh binding to PTCH involves co-receptors such as CAM-related/downregulated by oncogenes (CDO), brother of CDO (BOC) and growth arrest-specific 1 (GAS1) that form a multimolecular complex [93]. Shh signaling during the development of brain also requires low density lipoprotein receptor-related protein 2 (LRP2) as a coreceptor [94].

### Smo

During Shh signaling Smo undergoes a conformational switch [95] in its cytoplasmic domain from an inactive ‘closed’ conformation to an ‘open’ conformation, which is essential for Smo expression on plasma membrane and signal transduction. Smo activation also involves successive phosphorylation by protein kinase A (PKA), casein kinase Iα (CKIα), casein kinase II (CKII) and GPCR kinase 2 (GPRK2) [96, 97]. The extent of phosphorylation determines the strength of signaling [98]. Smo has been proposed to signal through G_αi_ to inhibit adenylyl cyclase and PKA activity [99].

### Gli family

In the absence of Shh ligand, Gli is phosphorylated by PKA that facilitates ensuing sequential phosphorylations by glycogen synthase kinase 3β (GSK3β) and CKI [100]. Phosphorylated Gli binds to β-transducin repeat-containing protein (βTrCP), which recruits S phase kinase-associated protein 1 (Skp1)-Cullin 1 (Cul1)-F-box protein (SCF) complex, an E3 ubiquitin ligase [100]. Subsequent ubiquitylation and proteasomal degradation of the C-terminal transactivation domain of Gli generates the ‘truncated’ Gli that represses gene expression in the nucleus. Shh signaling prohibits these post-translational modifications and degradation of Gli and, thus, ensures Gli stabilization and transcriptional activation of candidate genes. In the absence of Shh, Gli is also regulated by ‘suppressor of Fu’ (SUFU) [101], an adapter protein that sequesters Gli in the cytoplasm. Among the Gli family proteins, Gli2 is the major transcriptional activator [102] downstream of Shh signaling while Gli3 has primarily a repressor function [103]. Gli1 lacks repressor domain and serves a minor role to amplify Shh signaling [104].

### Signaling output

Shh signaling plays an important role during development as well as in adult tissue homeostasis. The output of Shh signaling can be extremely diverse depending on the tissue, which is determined by expression of varying set of target genes in response to concentration gradient of Shh [105–107]. For example, in the neural tube the genes encoding NK6 homeobox 1 (NKX6.1), oligodendrocyte transcription factor 2 (OLIG2) and NKX2.2 are expressed by progressively higher concentrations of Shh [105]. Shh plays an important role in the limb patterning from limb bud [107, 108] and development of nervous system from notochord and floor plate in the neural tube [105, 109]. Shh range of action can be as much as 300 microns in developing limb buds [108]. Shh maintains stem cells in several tissues such as hair follicle and hematopoietic system, and allows repair of tissues such as exocrine pancreas, prostate, and bladder after injury [106, 110].

Mutations in the components of Shh signaling or non-mutational dysregulation leads to cancer in organs such as skin (basal cell carcinoma) [111], brain (medulloblastoma) [112], pancreas, prostate, and breast [113]. Smo-antagonists such as vismodegib [114], sonidegib [115], saridegib [116] and glasdegib [117] have been investigated for the treatment of different tumor types including basal cell carcinoma (BCC), medulloblastoma, leukemias, cancers of pancreas, prostate, gastrointestinal tract, and breast [118]. Vismodegib has already received approval of US FDA for the treatment of advanced BCC [119].

### Non-canonical signaling

There are primarily two types of non-canonical signaling, which are non-genomic and often independent of Gli. Type I is Smo-independent and is a direct consequence of binding and inhibition of PTCH by Shh. PTCH-induced cell cycle arrest and apoptosis through cyclin B1 binding [120], and recruitment of caspase-3 [121], respectively, are prevented upon Shh binding. On the other hand, type II signaling is mediated by non-genomic actions of Gi proteins downstream of Smo and leads to activation of phosphoinositide 3-kinase (PI3K) [122], phospholipase Cγ (PLCγ) [123], and small GTPases, RhoA [124] and Rac1 [125]. For example, chemotaxis of fibroblasts induced by Shh is dependent on non-canonical Shh signaling to Rho GTPases [124], while PLCγ mediates Shh-induced calcium signaling in neural progenitor cells [123].

## WNT signaling

**Fig. 4:**
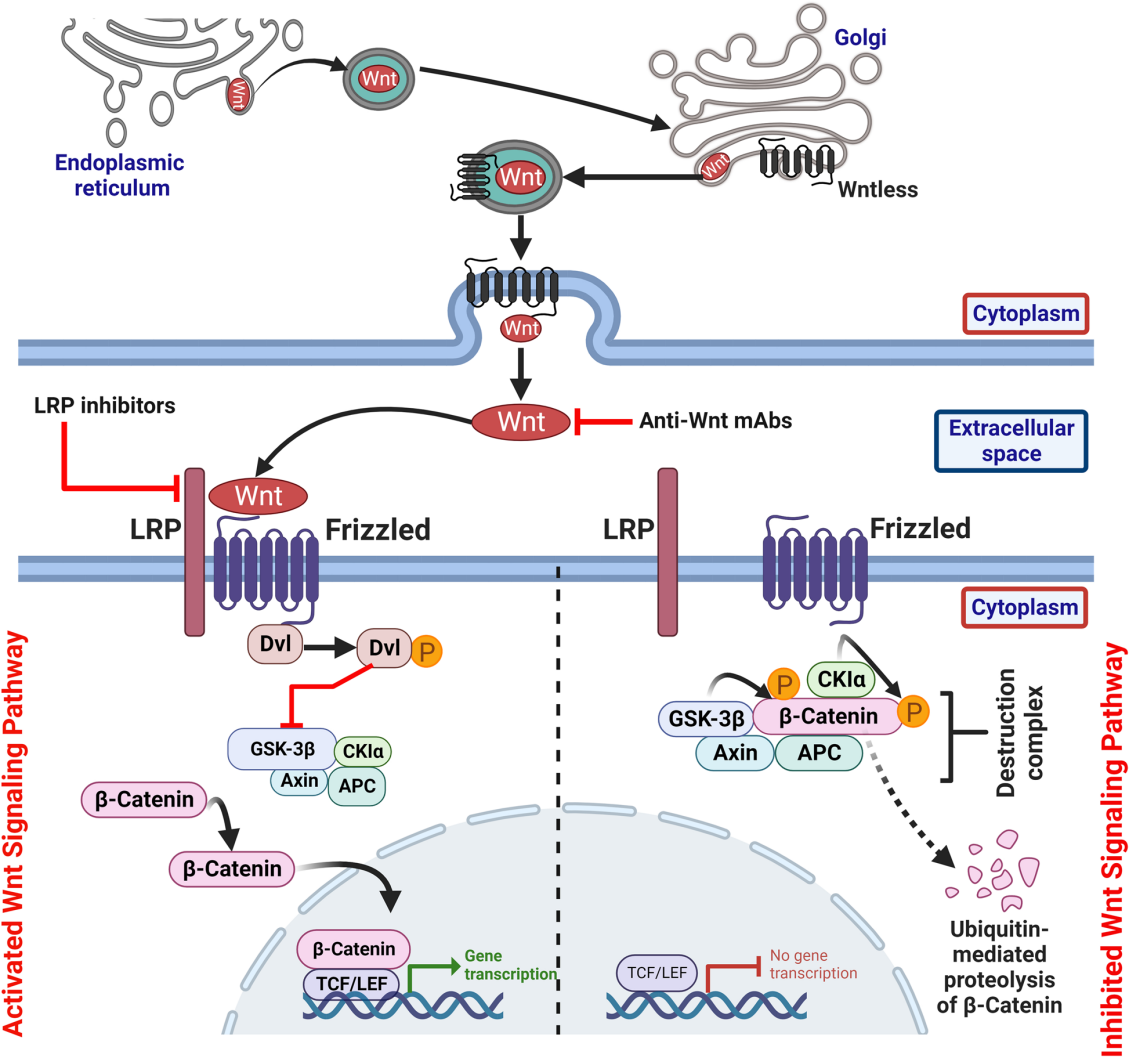
Canonical Wnt signaling. In the absence of Wnt ligand, the ‘off-state’ of signaling is characterized by the formation of a ‘destruction complex’ composed of casein kinase 1α (CK1α), glycogen synthase kinase-3β (GSK-3β), axis inhibition protein (AXIN) and adenomatous polyposis coli (APC) that phosphorylates, ubiquitinates and degrades β-catenin, which is the central effector of Wnt signaling pathway. Upon binding of Wnt ligand to Frizzled (FZD), the Wnt signaling pathway is switched on. The signaling cascade is initiated by recruitment of Dishevelled (DVL) to the plasma membrane. DVL, in turn, inhibits glycogen synthase kinase-3β (GSK-3β) activity, and thus prevents phosphorylation of β-catenin. Consequently, β-catenin is stabilized and translocated to the nucleus, where it regulates the expression of Wnt target genes. Trafficking of lipid modified Wnt (by porcupine) to the cell surface through Golgi network requires Wntless. Potential therapeutic agents targeting Wnt signaling include β-catenin antagonist, porcupine inhibitor and small molecule/mAb inhibitors of FZD, LRP5/6 and Wnt

Wnt derives its name from the wingless phenotype of *Drosophila* mutant with defective *‘Wingless’* (*Wg*) gene [8], in combination with its mammalian counterpart gene *‘int1’* [126]. Wnt signaling has an important role in regulating cell specification, polarity, and mitotic activity [127]. In the absence of Wnt ligand, the off-state of signaling is characterized by the formation of a ‘destruction complex’ (see below) that phosphorylates, ubiquitinates and degrades β-catenin, which is the central effector of Wnt signaling pathway. Upon binding of Wnt ligand to Frizzled (FZD), its cognate receptor, the Wnt signaling pathway is switched on. The signaling cascade is initiated by recruitment of Dishevelled (DVL), which is the key signal transducer of Wnt pathway, to the plasma membrane. DVL, in turn, inhibits glycogen synthase kinase-3β (GSK-3β) activity, and thus prevents phosphorylation of β-catenin. Consequently, β-catenin is stabilized and translocated to the nucleus, where it regulates the expression of Wnt target genes (Fig. 4) [127].

### Wnt

There are 19 Wnt paralogous genes in the mammalian genome [128]. Before their release from source cells each of these Wnts needs to undergo palmitoylation at N-terminal end catalyzed by Porcupine O-acyltransferase, an endoplasmic reticulum transmembrane acyltransferase, which is essential for binding of Wnt to FZD receptor [129, 130]. Subsequent trafficking of lipid-modified Wnt to the cell surface through Golgi network requires Wntless [131], another transmembrane protein. Paracrine signaling by the hydrophobic Wnt is facilitated by its spreading on cell surface with the help of transmembrane proteins known as glypicans [132]. Alternatively, Wnt is transported in vesicles along the actin-based cytonemes [133], delicate cytoplasmic extensions from the source cells, which are received by similar cytoplasmic projections from the Wnt target cells in the neighborhood.

### FZD and co-receptors

Like their ligands (Wnts), the FZD receptors, too, exhibit remarkable heterogeneity with 10 paralogous genes expressed in mammals [134]. The receptors are grouped into 4 clusters that bind to multiple Wnts and individual Wnts exhibit remarkable receptor promiscuity with cross-reactivity towards multiple FZD subtypes. Specific cellular response to Wnt subtypes is sometimes achieved through dependence on context-specific co-receptors such as Reck [135] during endothelial junction formation in CNS and EGFR [136] in hematopoietic stem and progenitor cell development.

### Signal transduction

In the signal ‘off’ state, β-catenin is sequentially phosphorylated by casein kinase 1α (CK1α) and GSK-3β in the presence of scaffolding proteins ‘axis inhibition protein’ (AXIN) and ‘adenomatous polyposis coli’ (APC) in a ‘destruction complex’ cluster [137]. Phosphorylated β-catenin is next polyubiquitinated by β-TrCP and SCF E3 ligase and subsequently fated to proteasomal degradation. Wnt-FZD binding initiates multiple phosphorylation events of the co-receptor LDL-receptor related protein 5/6 (LRP5/6) [138]. Simultaneously, DVL associates with FZD and undergoes oligomerization [139]. DVL oligomer, in turn, recruits ‘destruction complex’ (that comprises GSK-3β) to phosphorylated LRP5/6 forming a signalosome [140]. Formation of the signalosome is associated with inhibition of GSK-3β activity. In the absence of GSK-3β-mediated phosphorylation, β-catenin is stabilized and undergoes nuclear translocation leading to expression of target genes [141] (see below). Alternatively, β-catenin can interact with E-cadherin [142] at cell–cell junctions to mediate cell adhesion or localize to centrosomes to regulate mitosis [143].

### Signaling output

In nucleus β-catenin interacts with transcription factors belonging to T cell factor (TCF) [144] and lymphoid enhancer-binding factor (LEF) [145] families to regulate expression of Wnt target genes that dictate diverse cellular responses such as cell cycle progression, differentiation, and self-renewal [146]. Wnt/ β-catenin signaling is important for determining dorsoventral and anteroposterior body axes [147], maintenance of pluripotency of embryonic stem cells [148], and differentiation of embryonic stem cells into different germ layers during early embryonic development [149]. It is also important in homeostasis of various adult tissues such as intestine [150], bone [151] and skin [152] through maintenance of adult stem cells for tissue repair and regeneration [153]. Aberrant Wnt signaling is associated with degenerative diseases of the nervous [154, 155] and skeletal system [156], as well as cardiovascular [157] and metabolic [158, 159] diseases.

Mutations in Wnt signaling components or non-mutational dysregulation are associated with uncontrolled cell proliferation, epithelial mesenchymal transition (EMT) and metastasis in breast and colorectal cancer [160–162]. Molecules targeting Wnt pathway such as PRI-724 [163] (a β-catenin antagonist), LGK-974 [164] (a porcupine inhibitor) and Vantictumab [165] (an anti-FZD antibody) are in early clinical trials for the treatment of leukemia, melanoma, colorectal carcinoma and cancers of breast, lung, and pancreas [118]. In addition, two FDA-approved non-steroidal anti-inflammatory drugs (NSAIDs), namely sulindac [166], that also targets DVL, and celecoxib [167], that also inhibits β-catenin signaling, are under early clinical trials for their antineoplastic activity.

### Non-canonical signaling

There are two principal non-canonical Wnt signaling pathways:

i. The Wnt planar cell polarity pathway, which is activated by Wnt-FZD interaction independent of LRP, followed by recruitment of either DVL alone, or DVL and Disheveled-associated activator of morphogenesis 1 (DAAM 1). This triggers activation of Rho family of small GTPases, either Rac and RhoA, respectively, leading to RhoA-ROCK-dependent actin cytoskeletal rearrangements [168] and transcriptional activation of genes downstream of Rac-JNK-c-Jun pathway [169].
ii. The Wnt calcium pathway, which is also triggered by interaction of Wnt with FZD independent of LRP, leading to activation of Gq, phospholipase C-dependent release of inositol 1,4,5-trisphosphate (IP3)/ diacylglycerol (DAG), and spike in cytosolic calcium by release from ER stores [170]. Activation of this pathway leads to calcium-dependent signaling to multiple proteins including calcium/calmodulin-dependent protein kinases as well as protein kinase C.

## Morphogen signaling in Anucleate platelets

It has long been recognized that platelets have an abundant expression of proteins that primarily function within the nucleus including nuclear receptors [17] and transcription factors [16]. Initially their role in platelets was dismissed with a belief that most of these proteins were vestigial from bone marrow megakaryocytes from which circulating platelets are generated. However, as platelets expend energy to maintain expression of these proteins, they probably have some biological function. It is now established that these proteins have important non-genomic roles not just in platelets but also in other nucleated cells that can be described as ‘non-canonical’ [171–174]. Anucleate platelets with intact machinery for translational and post-translational regulation of proteins serve as an ideal model [175] to unravel the non-genomic roles of such proteins classically reserved for the nuclear niche.

While glucocorticoid receptor (GR) [176] and estrogen receptor (ER) [177] negatively regulate platelet activation responses, androgen receptor (AR) [178] signaling potentiates platelet aggregation. The underlying mechanisms for these non-genomic effects of nuclear hormone receptors are currently under active investigation. The non-genomic roles in platelets and the underlying mechanisms for transcription factors such as nuclear factor-κB (NF-κB) and peroxisome proliferator-activated receptor-γ (PPARγ) are more clearly established. NF-κB, for example, is activated in response to thrombin-induced protease activated receptor (PAR) signaling or collagen-induced signaling in platelets, which, in turn, instigates extracellular signal-regulated kinase (ERK)-dependent phospholipase A2 (PLA2) activation and TxA2 generation [179, 180]. NF-κB can also induce a negative feedback inhibition of platelet function through interaction with protein kinase A (PKA) [181]. A similar role for PPARγ in potentiating platelet activation has been identified. PPARγ associates with the tyrosine kinase Syk as well as the linker for activation of T‐cells (LAT) in collagen-stimulated platelets to promote activation of PLCγ2 and PI3K signaling [182]. PPARγ ligands inhibit platelet activation by preventing PPARγ association with Syk and LAT upon collagen stimulation [182].

As discussed earlier in preceding paragraphs, morphogen signaling comprises various pathways believed to operate only in nucleated cells leading to expression of specific genes involved in cell differentiation. However, and quite unexpectedly, over past few years components of these pathways are being discovered in platelets by multiple research groups including ours, which operate ‘non-canonically’ while remaining dedicated to the core functional proclivity of platelets towards hemostasis. Table 1 provides the relative mRNA expression of some of these components in platelets compared to other well-established signaling proteins involved in platelet activation [183]. We discuss here our own findings and those from other research groups investigating their functional relevance in platelet biology and possibility to harness components of these pathways as potential therapeutic targets in thrombo-occlusive pathologies.

**Table 1:** Relative expression of morphogen signaling gene transcripts in platelets compared to other well-established signaling molecules involved in platelet activation [183]

Signaling Molecule	Gene transcript level in platelets according to Blueprint database (log2fpkm)
Notch 2, 1 and 4	2.92, 2.71, 1,61
DLL4	0.3
RBPJ (CSL)	4.06
PTCH1	1.96
Smo	1.49
Gli 4, 3, 2, 1	1.43, 0.17, 0.15, 0.14
Wnt 16, 11, 5, 3	0.41, 2.23, 0.9, 0.07
Phospholipase C Beta 1, 2, 3, 4	1.27, 4.52, 2.03, 4.43
Phospholipase C Gamma 2, 1	4.07, 2.93
Akt 1, 2, 3	5.18, 4.85, 8.76
Syk	5.09
PAR 1, 4	8.31, 4.87
P2Y 12, 1, 2	8.85, 5.05, 1.12

### Notch signaling in platelets

Notch signaling inspires cell-fate decisions in differentiation of megakaryocytes (MK) from hematopoietic stem cells and eventual production of platelets from megakaryocytes [184]. Notch induces early megakaryocyte differentiation in mice [184] though it lacks similar influence in humans [185]. This underlines interspecies variations in Notch signaling pathway and MK development [186]. DLL-4-Notch axis has been shown to inhibit terminal MK differentiation and platelet production from human CD34 + cells by reducing the generation of mature MK and platelet-forming cells, possibly attributed to impaired transcriptional response in MKs [185].

Despite significant information in megakaryocytes, existence of functional Notch signaling in terminally differentiated platelets remained elusive until our group recently demonstrated significant expression of Notch1 and its ligand, the Delta-like ligand (DLL)-4, as well as their respective transcripts, in human platelets [20]. Platelets have a functional Notch signaling pathway as evidenced by proteolytic cleavage of Notch1 receptor to NICD in a γ-secretase-dependent fashion upon exposure of platelets to exogenous DLL-4. γ-secretase activity in platelets is also known to produce all the proteolytic products of amyloid precursor protein (APP) including soluble APPα, soluble APPβ, and amyloid β) [187, 188].

DLL-4-Notch1-NICD signaling axis is of relevance to platelet function as DLL-4 on its own induces platelet responses characteristic of activation [20]. This includes high-affinity fibrinogen binding through conformationally active integrin receptor, secretion of dense and alpha granule contents, shedding of extracellular vesicles and platelet-leukocyte interaction, all of which promote thrombus formation at the site of vessel injury. DLL-4 also provokes a rise in intracellular calcium and tyrosine phosphorylation of proteins, which are both considered hallmark signaling events proximate to platelet activation. Considering these evidence DLL-4 possibly induces platelet activation much like conventional platelet agonists.

The cellular response to canonical Notch signaling is a consequence of the actions of NICD/CSL within the nuclear niche, which cannot operate in an anucleate cell such as the platelet, suggesting non-canonical Notch signaling in platelets. In concurrence, DLL-4 induces non-canonical PI3K-AKT signaling in platelets in a γ-secretase-dependent manner. PI3K-AKT pathway, in turn, mediates DLL-4-induced platelet activation.

Both synthesis and surface expression of DLL-4 are significantly enhanced upon platelet stimulation with thrombin, a physiological agonist, signifying a role for endogenous DLL-4 in platelet activation during thrombus formation, possibly through juxtacrine DLL-4-Notch1 signaling between closely packed neighboring platelets within the thrombus. This hypothesis is supported by three lines of evidence. First, the presence of exogenous DLL-4 potentiates thrombin-induced platelet activation and aggregation. Second, DAPT, a γ-secretase inhibitor, significantly blights thrombin-induced platelet activation and aggregation without the presence of exogenous DLL-4. DAPT pre-treatment also inhibits human platelet thrombus formation on immobilized collagen under arterial shear ex vivo as well as arterial thrombosis in mice. Third, the presence of an anti-DLL-4 antibody, which prevents DLL-4-Notch1 interaction between adjacent platelets, significantly compromises thrombin-induced platelet aggregation.

**Fig. 5:**
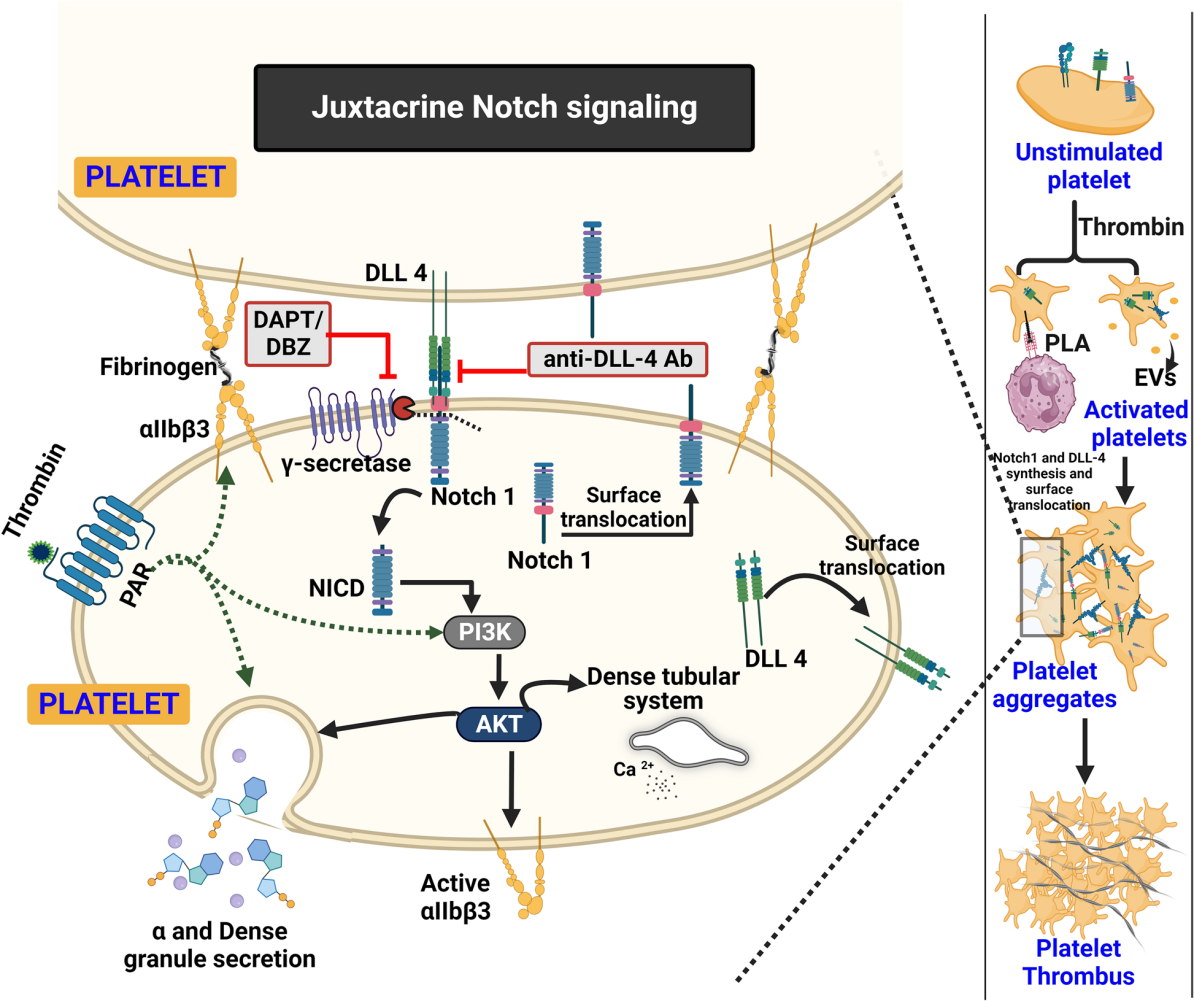
Scheme depicting the role of Notch signaling in stimulated platelets. There is juxtacrine interaction between DLL-4 and Notch1 expressed on surfaces of agonist-stimulated platelets that remain in close proximity within platelet aggregates. This leads to Notch-mediated non-canonical PI3K-AKT signaling in the partner platelet potentiating thrombin-induced platelet activation responses and thrombus formation, which can be blocked by anti-DLL-4 antibody or inhibitors of γ-secretase (DAPT or DBZ)

In summary, the study presents compelling evidence in support of non-canonical Notch signaling that propagates in juxtacrine manner within platelet aggregates and synergizes with physiological agonists to generate occlusive intramural thrombi at the site of vessel injury (Fig. 5) [20]. Molecules targeting Notch signaling including the γ-secretase inhibitors and anti-DLL-4 antibody, which are currently being investigated in clinical trials for cancer, can be promising candidates for antiplatelet and antithrombotic therapies.

### Shh signaling in platelets

Ever since the elucidation of Wnt signaling in platelets by Steele et al. in 2009 [189], there have been attempts to unravel other morphogen signaling pathways in platelets including Shh. A series of studies by our group established expression of various components of Shh pathway in platelets, such as the PTCH receptor [190], the transcription factor Gli [190] and more recently the Shh ligand [18], raising the possibility of a functional Hedgehog signaling operative in platelets. Keeping with this, platelets synthesize Shh from pre-existing mRNAs when challenged with physiological agonists, mobilize it for surface expression and release on microvesicles, thus alluding to its putative role in platelet activation [18]. Shh synthesis, plasma membrane translocation and shedding on extracellular vesicles are all increased upon thrombin stimulation of platelets. Shh ligand, in turn, evokes a wave of non-canonical signaling in platelets leading to activation of small GTPase RhoA and phosphorylation of myosin light chain (MLC) in AMP-activated protein kinase (AMPK)-dependent manner. While the canonical Shh signaling cascade acts through Gli family transcription factors, our study revealed a distinct non-canonical Shh signaling mediated by RhoA/AMPK that reinforces platelet activation and arterial thrombosis. In support, Smo-antagonist cyclopamine significantly blunts thrombin-induced activation of RhoA and AMPK signaling axes in platelets. Cyclopamine, as well as the FDA-approved Smo-inhibitor vismodegib, significantly decrease aggregation of platelets in response to a multitude of agonists as well as restrict their fibrinogen binding, integrin activation, secretion of dense and alpha granule contents, shedding of extracellular vesicles and spreading on immobilized matrix, all of which promote platelet thrombogenicity. In consistence, both molecules effectively inhibit platelet thrombus formation on collagen under arterial shear ex vivo as well as arterial thrombosis in mice. This underscores the role of feed-forward paracrine/juxtacrine inputs from Shh in consolidation of thrombus [18].

Interestingly, our group also discovered that pre-treatment of platelets with Shh prevents ABT-737-induced caspase-3 activation, mitochondrial depolarization, and PS exposure [190]. ABT-737 is a Bcl-2 homology-3 domain (BH3)**-**mimetic that inhibits Bcl-2/Bcl-xL, thus leading to Bax/Bak-dependent cell death by intrinsic apoptotic pathway. Incidentally, Bcl-x_L_ and Bak constitute an ‘internal timer’ that determines platelet lifespan in the circulation [191]. We also found Shh to protect against thrombin-induced apoptosis-like changes in platelets [190]. We posit a role for Shh signaling in promoting platelet survival.

**Fig. 6:**
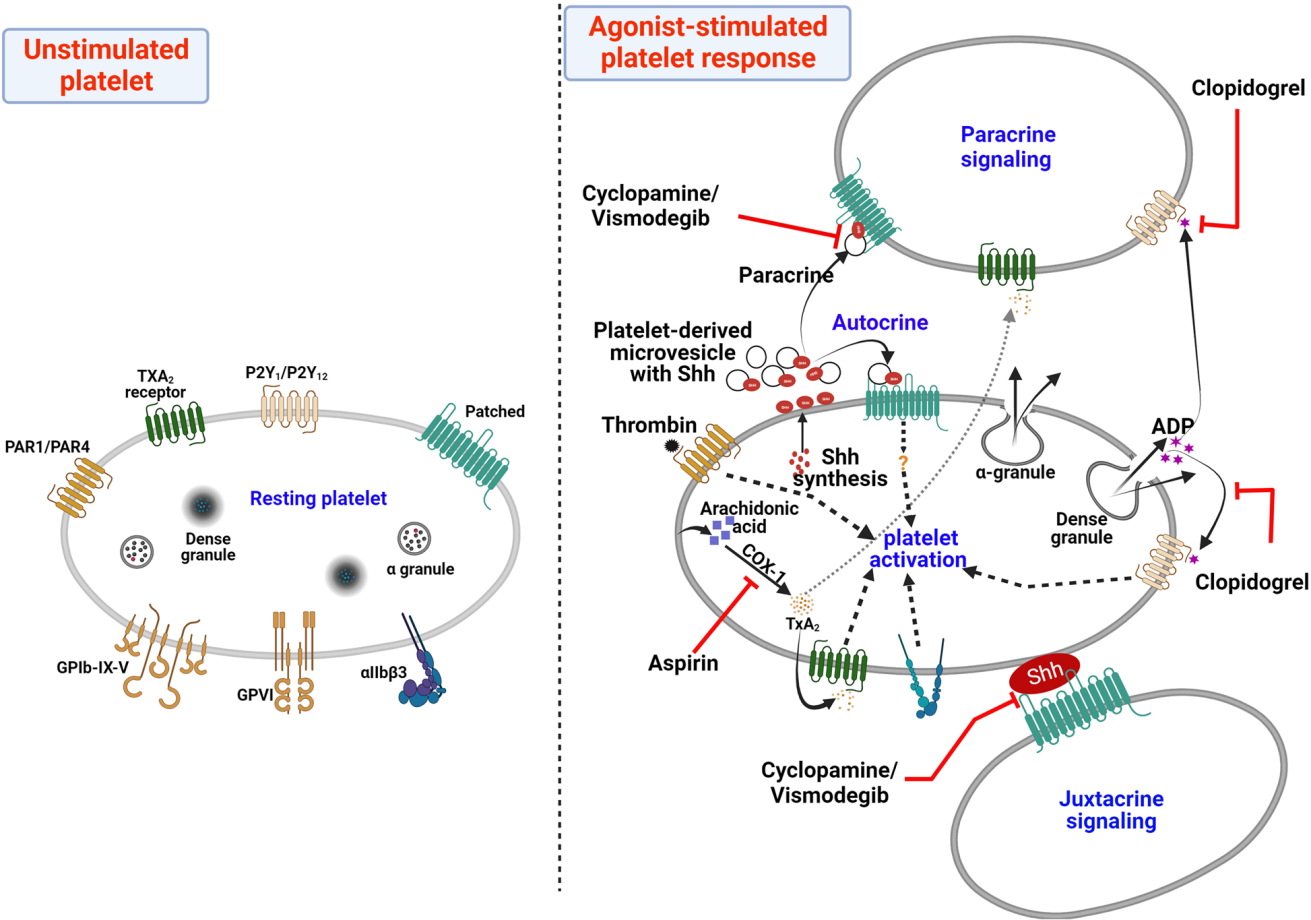
Scheme depicting the role of Shh signaling in stimulated platelets. Short-range autocrine/juxtacrine/paracrine signaling by Shh amplifies thrombin-induced platelet activation responses through non-canonical activation of RhoA and AMP-activated protein kinase (AMPK) in a feed-froward loop akin to those involving ADP and TxA2. Shh antagonists (cyclopamine/vismodegib), cyclooxygenase inhibitors (aspirin), and P2Y12 antagonists (clopidogrel) target these feed-forward loops to limit platelet activity

In summary, these observations support a feed-forward loop of platelet stimulation established locally by Shh, like ADP and thromboxane A2, through non-canonical paracrine/juxtacrine short-range signaling within a growing thrombus. Shh amplifies agonist-driven platelet agonists while allowing activated platelets to thrive and thereby contributes significantly to stability of occlusive arterial thrombus (Fig. 6) [18]. Thus, targeting Shh signaling in platelets could be an effective antithrombotic strategy.

### Wnt signaling in platelets

One of the earliest elucidations of morphogen signaling in platelets was the discovery by Steele et al. in 2009 that platelets express components of Wnt signaling pathway including the receptor Fzd6/4-LRP5/8, the ligand Wnt3a, the transducer Dvl-2, the destruction complex composed of CK1, GSK3β, Axin-1, FRAT-1 and APC, as well as the effector protein β-catenin [189]. Exogenous Wnt3a negatively regulates agonist-induced platelet responses, particularly integrin activation and ensuing aggregation through fibrinogen bridging, dense granule secretion, shape change and static adhesion on fibrinogen. Further, stimulated platelets were known to secrete Wnt3a into their surrounding and platelets lacking Fzd6 with deficient Wnt signaling are hyperresponsive to moderate doses of thrombin. These findings strongly suggest that platelet-derived Wnt3a can potentially exert a negative feedback regulation of platelet activation [189], contrasting the impact of Shh and Notch signaling pathways on platelet activity elucidated years later.

Exogenous Wnt3a induces canonical signaling in both resting and agonist-stimulated platelets leading to impaired β-catenin phosphorylation by GSK3β, which would lead to β-catenin stabilization [189]. However, there is lack of evidence to support β-catenin accumulation in platelets in response to Wnt3a exposure. This can be attributed to considerable expression of β-catenin in resting platelets as reported by us [192] possibly due to a lack of constitutive proteolytic degradation. However, there is substantial cleavage of β-catenin through ubiquitin–proteasome system as well as calpain activity upon sustained platelet aggregation induced by thrombin [192]. Remarkably, calpain-mediated β-catenin degradation in aggregating platelets is not dependent on GSK3β-driven phosphorylation but appears to be provoked by PKC activity through integrin outside-in signaling [192]. Although β-catenin stabilization by Wnt3a cannot be ruled out in thrombin-stimulated platelets, this seems unlikely due to the calpain-dependent degradation. Hence, is it reasonable to posit that canonical Wnt signaling in platelets does not have any functional outcome due to two consequential reasons: 1) Wnt3a does not stabilize β-catenin in stimulated platelets, 2) β-catenin cannot exercise its transcriptional regulation of proteins in anucleate platelets.

**Fig. 7:**
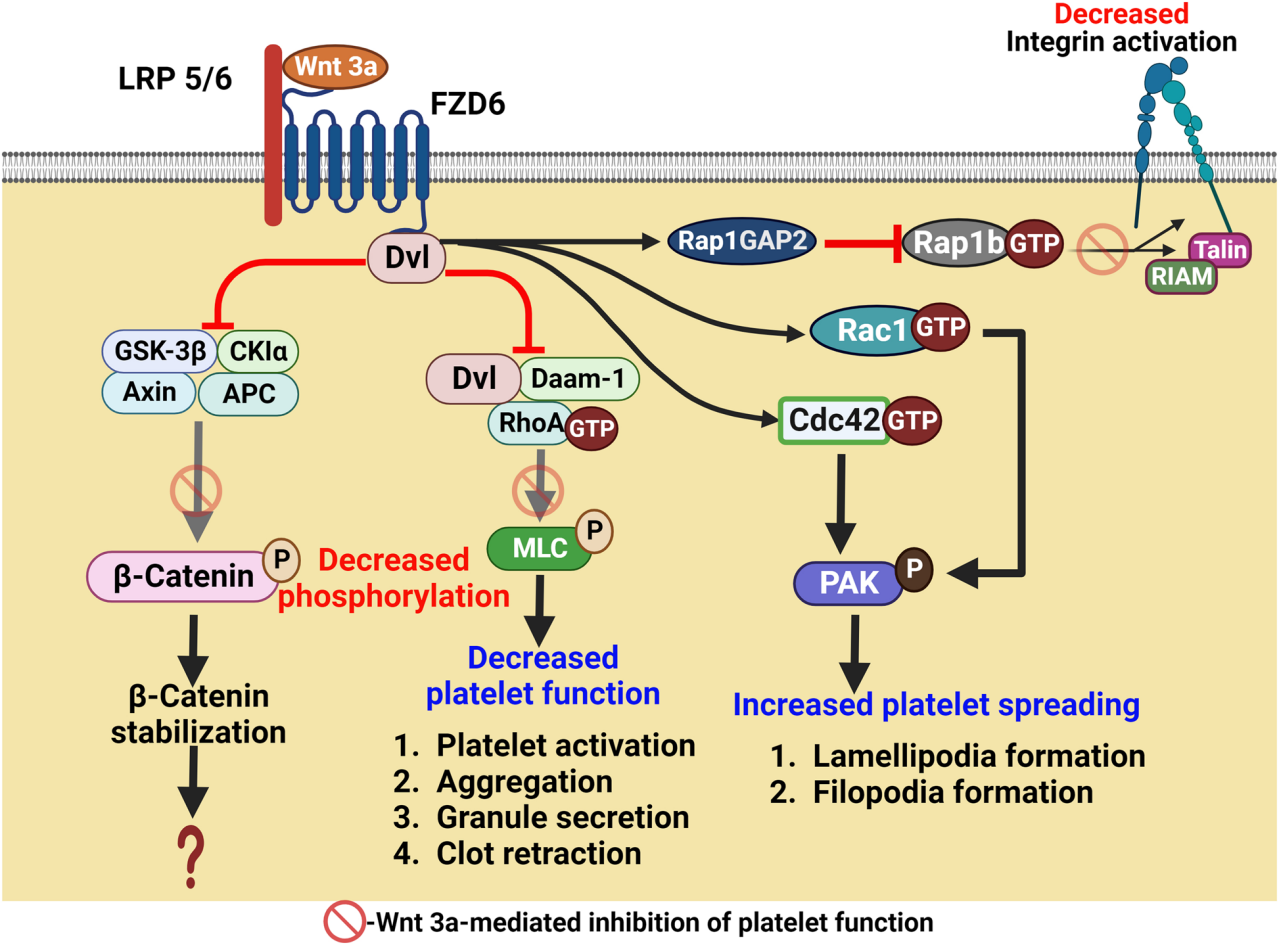
Scheme depicting the role of Wnt signaling in stimulated platelets. Wnt3a induces non-canonical signaling in platelets leading to inhibition of RhoA and Rap1b activities as well as activation of Rac1 and Cdc42. Thus, Wnt3a negatively regulates most agonist-induced platelet activation responses but increases platelet spreading on collagen matrix. Wnt3a also induces canonical signaling in both resting and agonist-stimulated platelets leading to impaired β-catenin phosphorylation by GSK3β that could lead to β-catenin stabilization whose implications however are unknown

It is safe here to surmise that, Wnt3a ushers non-genomic non-canonical signaling in platelets that leads to impaired platelet activation. Although Wnt3a does not affect calcium signaling in platelets, there is considerable evidence in support of regulation of activities of multiple small GTPases critical to platelet function. Wnt3a negatively regulates RhoA-GTP signaling mediated by Dvl-Daam1 interaction in stimulated platelets [19]. GTP-bound RhoA prompts actomyosin contraction in stimulated platelets underlying shape change, aggregation, and granule secretion. Wnt3a promotes Rap1GAP2-dependent downregulation of Rap1-GTP that plays a critical role in conformational activation of platelet integrins α_IIb_β_3_. Counterintuitively, Wnt3a boosts the expression of CDC42-GTP and Rac1-GTP that are known to promote formation of actin-rich structures like filopodia and lamellipodia, respectively, in platelets adhered on immobilized matrix. In agreement, Wnt3a accelerates platelet spreading on fibrinogen despite negative regulation of other platelet activation responses. Most of the effects of Wnt pathway on platelet function are likely mediated by non-canonical signaling through small GTPases, RhoA, Rap1b, CDC42 and Rac1 (Fig. 7) [19].

## Conclusion

**Fig. 8:**
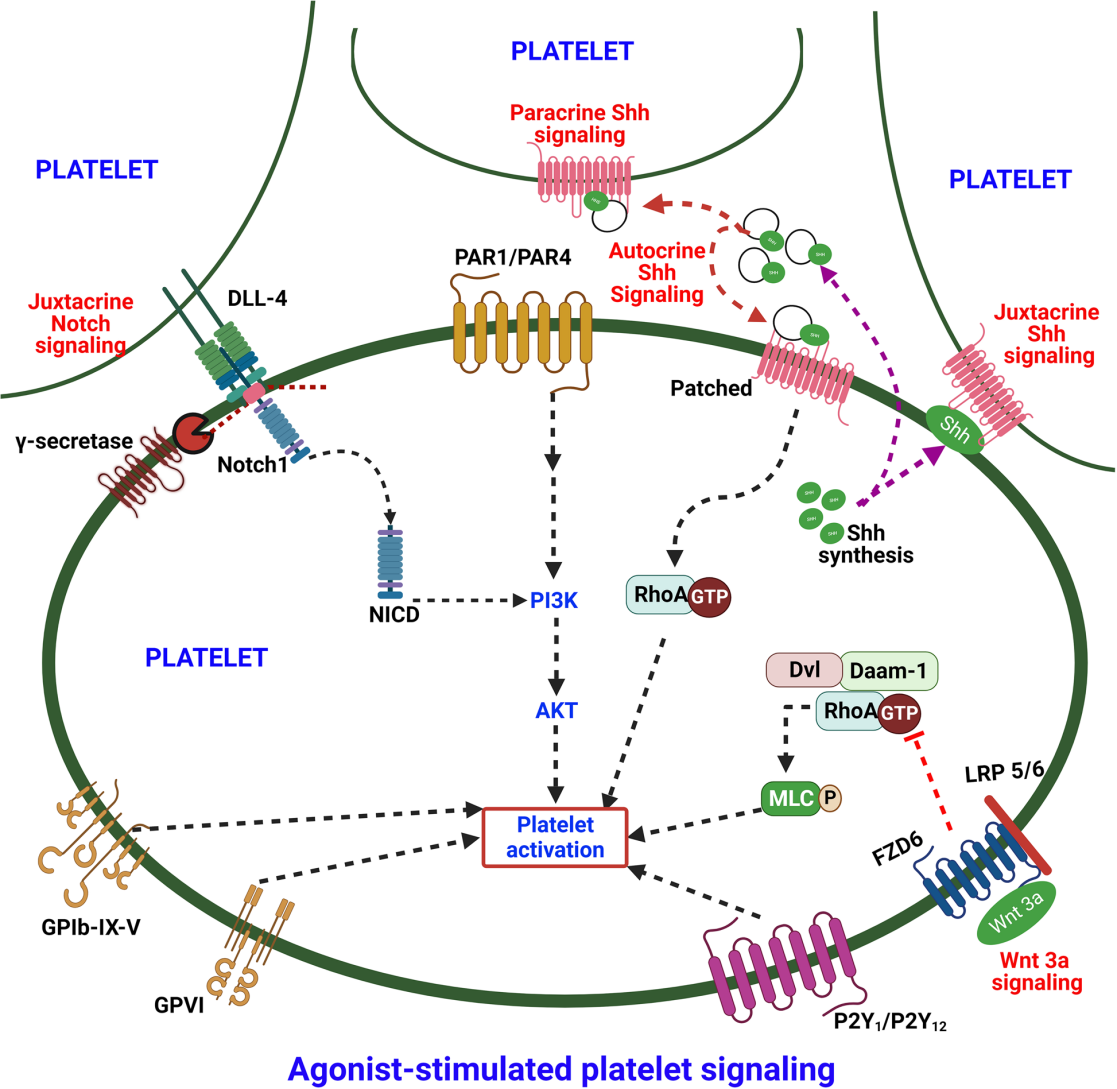
Scheme depicting the role of morphogen signaling in stimulated platelets. Shh and DLL-4-Notch1 from agonist-stimulated platelets establish feed-forward loops of autocrine/juxtacrine/paracrine non-canonical signaling that help perpetuate agonist-induced platelet activation. On the other hand, non-canonical Wnt signaling is part of a negative feedback loop for restricting platelet activation

Anucleate platelets express functional morphogen pathways that serve to modulate platelet function and survival in circulation through non-canonical non-genomic signaling (Fig. 8). Shh and DLL-4-Notch1 from agonist-stimulated platelets establish feed-forward loops of autocrine/juxtacrine/paracrine non-canonical signaling that helps perpetuate thrombosis much like conventional secondary mediators of platelet activation like ADP and thromboxane A2 (TxA2). Drugs such as aspirin and clopidogrel that respectively target pathways mediated by TxA2 and ADP have been among the most widely employed antiplatelet drugs in clinics. Hence, molecules targeting Shh and Notch signaling pathway, some of which are already in clinical use or under trial for other applications like anticancer therapeutic, could be effective antithrombotic agents either as monotherapy or in combination with conventional antiplatelet drugs. On the other hand, non-canonical Wnt signaling is part of a negative feedback loop for restricting platelet activation and possibly limiting thrombus growth in sync with other platelet regulators such as prostacyclin and nitric oxide. While the influence of Wnt signaling in platelets in regulating thrombosis and hemostasis in vivo remains to be further ascertained, it could be one of the novel antithrombotic therapeutic avenues to investigate in future. However, we need to exercise utmost caution before considering drugs targeting morphogen signaling for clinical use as antiplatelet or antithrombotic drugs due to an abundant potential for off-target effects and adverse reactions.

Despite the available literature establishing role of morphogen signaling in platelet biology, several aspects remain largely unexplored. There is evidence for considerable crosstalk between different morphogen signaling pathways in nucleated cells. However, its existence and functional relevance to thrombogenicity remain to be deciphered in platelets. A clear understanding of putative interactions between the various pathways in stimulated platelets will help design more effective morphogen-targeting therapeutic strategies for disorders of platelet function. There could be multiple endogenous sources for morphogens in the circulation apart from the activated platelets themselves that need to be unraveled. Further, they can be altered in various physiological or disease states leading to platelet hyperactivity and thrombosis potentially serving as biomarkers and pharmacological targets in these disorders. In conclusion, morphogen signaling is critical for optimal platelet function and is growing to be one of the major focus areas of research for understanding platelet physiology as well as its therapeutic targeting. Several drugs targeting the morphogen signaling pathways are being evaluated in clinical trials for cancer. Platelets not only mediate thrombosis and prevent bleeding the risks of each being elevated in cancer patients, but they are also crucial for progression of cancer itself through promoting angiogenesis and metastasis. Thus, understanding the potential impact of targeting morphogen signaling could have on platelets would be of paramount importance in immediate future.
